# The Impact of Mechanical Bowel Preparation and Oral Antibiotics in Colorectal Cancer Surgery (MECCA Study): A Prospective Randomized Clinical Trial

**DOI:** 10.3390/jcm13041162

**Published:** 2024-02-19

**Authors:** Maximos Frountzas, Victoria Michalopoulou, Georgia Georgiou, Despoina Kanata, Maria Matiatou, Despina Kimpizi, Georgia Matthaiou, Spilios Spiliotopoulos, Dimitrios Vouros, Konstantinos G. Toutouzas, George E. Theodoropoulos

**Affiliations:** Colorectal Unit, First Propaedeutic Department of Surgery, Hippocration General Hospital, School of Medicine, National and Kapodistrian University of Athens, 11527 Athens, Greecedespkanata1@gmail.com (D.K.); despinakimpizi@yahoo.com (D.K.); sspi1924@gmail.com (S.S.); tousur@hotmail.com (K.G.T.);

**Keywords:** preparation, antibiotics, colorectal, cancer, surgery

## Abstract

**Background:** Colorectal cancer surgery has been associated with surgical site infections (SSIs), leading to an increase in postoperative morbidity, length of stay and total cost. The aim of the present randomized study was to investigate the relationship between the preoperative administration of oral antibiotic therapy and SSI rate, as well as other postoperative outcomes in patients undergoing colorectal cancer surgery. **Material and Methods:** Patients who underwent colorectal cancer surgery in a university surgical department were included in the present study. Patients were randomized into two groups using the “block randomization” method. The intervention group received three doses of 400 mg rifaximin and one dose of 500 mg metronidazole per os, as well as mechanical bowel preparation the day before surgery. The control group underwent only mechanical bowel preparation the day before surgery. The study has been registered in ClinicalTrials.gov (NCT03563586). **Results:** Two hundred and five patients were finally included in the present study, 97 of whom received preoperative antibiotic therapy per os (intervention group). Patients of this group demonstrated a significantly lower SSI rate compared with patients who did not receive preoperative antibiotic therapy (7% vs. 16%, *p* = 0.049). However, preoperative antibiotic administration was not correlated with any other postoperative outcome (anastomotic leak, overall complications, readmissions, length of stay). **Conclusions:** Preoperative antibiotic therapy in combination with mechanical bowel preparation seemed to be correlated with a lower SSI rate after colorectal cancer surgery.

## 1. Introduction

Colorectal cancer stands as the second most common cause of cancer-related death worldwide [1,2]. However, surgical intervention continues to be the primary treatment modality [3]. The preoperative preparation of an elective colorectal cancer (CRC) operation and the postoperative events predominantly affect its therapeutic purpose. The most prevalent complications after elective CRC surgery include anastomotic leakage (AL), with an incidence of 3–30%, and surgical site infection (SSI), which ranges from 5 to 30% [4,5]. The significant morbidity and high cost associated with SSI and AL after colorectal cancer surgery have prompted a focused initiative to identify strategies for reducing their occurrence [6].

While SSIs have been associated with multiple patient-related factors, such as obesity [7] or smoking [8], there are also surgery-related factors, including surgical procedures, surgeons’ techniques, mechanical bowel preparation (MBP) or antibiotics administration that could affect SSI rate after CRC surgery [9]. Various preoperative antibiotic regimens and combinations with preparation agents, which aimed at reducing their incidence, have been explored. Existing guidelines suggest that the combination of preoperative MBP with oral antibiotics (oAB), such as macrolides, is more effective in preventing SSIs compared with other antibiotic regimens or no bowel preparation (NBP) [10]. However, there is no conclusive evidence supporting the absolute effectiveness of combined preoperative MBP and alternative antibiotic regimens in diminishing the adverse effects of macrolides, such as diarrhea, nausea and abdominal pain [11]. The absence of robust evidence is even more pronounced when considering AL and other postoperative complications [12].

Due to the impact of postoperative complications on patients’ quality of life and the dynamic nature of CRC surgery, a significant controversy surrounds the most effective preoperative interventions for elective CRC surgery [13]. Beyond the traditional approaches of using MBP alone or combined with macrolide oAB, alternative methods, such as using different antibiotic regimens or employing oAB alone without MBP, are emerging. This suggests that the application of MBP may be more of a customary practice than one grounded in evidence [14]. In light of this background, there is a pressing need for randomized controlled trials to be conducted. These trials are essential to contributing crucial insights and addressing the existing uncertainties surrounding contemporary preoperative practices in elective CRC surgery.

The aim of the MECCA trial was to investigate the relationship between the administration of alternative preoperative oAB in conjunction with MBP compared with MBP alone and their impact on the incidence of SSIs, as well as other postoperative complications of elective CRC surgeries, such as AL, 30-day mortality, readmission rate, hospital length of stay and postoperative ileus.

## 2. Methods

### 2.1. Study Design and Participants

The present study included prospectively enrolled patients who underwent elective surgery due to colorectal cancer at the Colorectal Unit of a university surgical department in a tertiary hospital from 2018 until 2021. Inclusion criteria were adult patients, elective operation, open approach and preoperative diagnosis of colorectal cancer. Exclusion criteria were inoperability, emergency admissions, multi-organ excision, stoma creation, contraindication for preoperative administration of oAB or MBP and perioperative septic condition that required systemic antibiotic administration.

Randomization was performed using the “block randomization” technique, which has the advantage of increasing the comparability between groups by keeping the ratio of the number of subjects between groups almost the same. Moreover, it ensures that the number of subjects between groups is basically equal, maximizing the effectiveness of clinical trials as the standard error of the treatment-effect estimate is decreased, which affords big rewards in scientific accuracy and credibility [15]. Using the “block randomization” technique, included patients were divided into two distinct groups following a computer algorithm [16]. The intervention group (IG) received three doses of 400 mg rifaximin and a single dose of 500 mg metronidazole orally in combination with two doses of orally administered sodium phosphate solution (MBP) the day before surgery. On the other hand, the control group (CG) received only two doses of orally administered MBP the day before surgery. Patients in both groups received a single dose of 2 g cefoxitin and a single dose of 500 mg metronidazole intravenously administered 1 h before anesthesia induction. Antibiotic administration was stopped postoperatively. Both investigators and patients were unaware of the group in which they had been included (double-blind protocol).

### 2.2. Ethical Approvals

This study was approved by the Ethical Committee of the ‘’Hippocration’’ General Hospital of Athens (Ref. No.: 11-08/19-03-2018). It was conducted in compliance with the Declaration of Helsinki guidelines about ethical principles for medical research involving human subjects. A written informed consent was obtained by all patients before participation in the study. The trial has been registered on ClinicalTrials.gov under the identifier NCT03563586.

### 2.3. Study Parameters and Outcomes

Demographic characteristics of the included patients, such as age, gender and tumor location, were reported. In addition, past medical history parameters, such as hypertension, diabetes, heart arrhythmia, ischemic heart disease, renal dysfunction, chronic obstructive pulmonary disease (COPD) and past abdominal operation, were documented.

The primary study outcome was surgical site infection (SSI) according to the Centers for Disease Control (CDC) classification in 30 postoperative days [17]. Secondary outcomes were anastomotic leak (AL), overall postoperative complications during 30 postoperative days, readmissions and hospital length of stay (LOS).

### 2.4. Statistical Analysis

Statistical analysis was conducted for patients who finally reached follow-up after the exclusion of some other patients due to several reasons (per protocol analysis). The Kolmogorov–Smirnov test was used to investigate the normality of distributions among quantitative variables. Mean values and standard deviations (SD) were used for normally distributed outcomes, while absolute (N) and relative (%) frequencies were used to describe qualitative variables. Comparisons of proportions were performed using Pearson’s χ^2^ test, and comparisons of quantitative variables between two groups were conducted using the non-parametric Mann–Whitney U test.

Independent factors related to SSI, anastomotic leak, overall complications, readmissions and length of stay were investigated using logistic regression analyses that were performed using the stepwise inclusion/exclusion procedure and odds ratios (OR) along with their 95% confidence intervals (95% CI) were calculated. Significance levels were two-sided, and the statistical significance level was set at *p* = 0.05. A post hoc power analysis was conducted, considering the significance level (alpha) at 0.05.

## 3. Results

### 3.1. Included Patients

**Figure 1:**
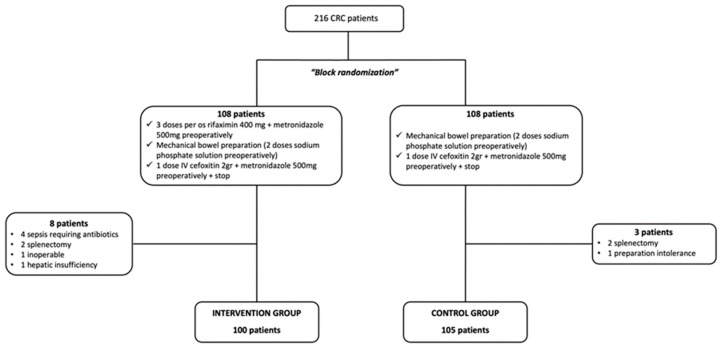
Study flowchart.

The present study initially included 216 patients who underwent elective surgery due to colorectal cancer. After the randomization process, two groups (intervention and control) of 108 patients were formed. However, eight patients were excluded from the intervention group (IG) due to sepsis that required prolonged perioperative antibiotic administration (four patients), splenectomy (two patients), hepatic insufficiency (one patient) and inoperable tumor (one patient). Moreover, three patients were excluded from the control group (CG) due to splenectomy (two patients) and MBP intolerance (one patient). Therefore, 100 patients of the IG and 105 patients of the CG reached follow-up and were included in the analysis (Figure 1).

### 3.2. Patient Characteristics and Postoperative Outcomes

The mean age of the included patients was 70 ± 11 years old (Table 1). The male patients were 118 (58%), and the female patients were 85 (42%). The tumor was located at the ascending colon in 36 patients (18%), at the transverse colon in 46 patients (22%), at the descending colon in 25 patients (12%), at the sigmoid colon in 56 patients (27%) and at the rectum in 42 patients (21%). In addition, 24 patients (12%) suffered from recurrent colorectal cancer. Hypertension was the most prominent among comorbidities, as 100 patients (49%) suffered from it. Following comorbidities included diabetes (21%), ischemic heart disease (12%), COPD (8%), heart arrhythmia (7%) and renal dysfunction (2%). Past abdominal operations were reported in 103 patients (50%). Eight patients (8%) in the intervention group and 11 patients (10%) in the control group had a loop ileostomy.

A surgical site infection was observed in 24 patients (12%), and anastomotic leakage was detected in 11 patients (5%). Furthermore, 15 patients (7%) were readmitted to the hospital after discharge and 155 patients (76%) were hospitalized for more than 7 days. Finally, the overall complication rate reached 22% (Table 1). The most common postoperative complications were ileus (10 patients), bleeding (5 patients) and acute ischemic heart disease (4 patients).

### 3.3. Univariable Analysis

Preoperative oral administration of rifaximin and metronidazole in combination with MBP was significantly associated with lower SSI rates in patients undergoing elective surgery due to colorectal cancer (7% vs. 16%, *p* = 0.049). However, similar findings were not observed in terms of anastomotic leak in such patients (4% vs. 7%, *p* = 0.447). Moreover, overall complications were similar among patients who received the preoperative oral administration of rifaximin and metronidazole in combination with MBP and patients who received only MBP (18% vs. 28%, *p* = 0.095). Finally, readmission rates were similar between the two study groups (5% vs. 10%, *p* = 0.237), as well as length of stay over 7 days (77% vs. 76%, *p* = 0.955) (Table 2).

### 3.4. Multivariable Analysis

Multivariable analysis revealed no significant correlations between SSI rates and overall postoperative complications in patients undergoing elective surgery due to colorectal cancer. However, anastomotic leak was significantly associated with ischemic heart disease (OR 11.1, 95% CI 1.6–75.4, *p* = 0.013). In addition, readmission rates were associated with COPD (OR 8.1, 95% CI 1.8–35.3, *p* = 0.006), and hospitalization over 7 days was correlated to a history of past operations (OR 2.1, 95% CI 1.1–4.4, *p* = 0.039).

## 4. Discussion

The present clinical trial demonstrated a notable reduction in the incidence of SSIs when using preoperative oAB administration in conjunction with MBP, as opposed to relying solely on preoperative MBP. This outcome underscores the efficacy of the combined intervention in mitigating the risk of SSIs in the context of elective colorectal cancer (CRC) surgery. Our comprehensive analysis did not reveal any statistically significant disparities in the occurrence of ALs or any other postoperative complications subsequent to elective CRC surgery. In addition, the impact of past medical history of patients undergoing elective surgery due to CRC on their postoperative results was outlined, as patients with ischemic heart disease presented a higher risk for anastomotic leak, COPD led to higher readmission rates and past abdominal surgery was associated to increased risk for hospital length of stay over 7 days.

The efficacy of preoperative oAB combined with MBP in reducing postoperative complications following elective CRC surgeries has been a matter of debate, with numerous clinical trials and meta-analyses in the literature attempting to provide clarity on this topic. The majority of conducted studies, including the present one, suggest a substantial decrease in SSIs with the combination of MBP and oAB, usually macrolides, aligning with current preoperative preparation guidelines [10,18,19,20]. However, conflicting data exist regarding its impact on other complications, such as AL, mortality, hospital length of stay (LOS) and ileus. Morris et al. [21], Futier et al. [22] and Lee et al. [23] indicated a decrease in SSIs and readmission rates with combined preoperative MBP and oAB, whereas no significant benefits were observed in other postoperative complications [24,25].

Following meta-analyses [26,27,28,29] have affirmed the beneficial impact of preoperative MBP plus oAB on SSI rate. Furthermore, those analyses have shown an association of MBP plus oAB with AL, mortality and ileus while suggesting the conduction of more extensive studies and randomized trials before establishing a robust conclusion. While our study reveals no significant difference in the incidence of AL or other postoperative complications with the use of MBP plus oAB compared with MBP alone, numerous studies exist that demonstrate the contrary. Ambe et al., in a prospective study [30], illustrated the association between the use of MBP plus oAB and a decreased risk of AL. This finding is supported by Willis et al. [4], although caution is warranted due to the relatively low overall incidence of postoperative AL. In a more recent literature context, Lei et al. [31] proposed a region-specific impact of preoperative MBP plus oAB in SSI prophylaxis, suggesting that the effectiveness is notably greater for elective left-sided CRC surgeries. On the other hand, Koskenvuo et al. [32], in the randomized, single-blinded MOBILE trial, questioned the reduction in SSI rates with the administration of MBP plus oAB compared with no bowel preparation (NBP), prompting a reevaluation of the existing guidelines [10].

Although the combination of MBP and oAB appears to be the most favorable option based on the aforementioned information, uncertainties persist regarding the efficacy and necessity of MBP in isolation. Conventional practice dictates the dogmatic use of MBP before CRC surgery, with the primary goal of reducing fecal mass and bacterial count. This aims to decrease rates of SSIs and ALs while facilitating dissection and endoscopic evaluation [33]. Nevertheless, an increasing number of studies challenge its effectiveness, suggesting that the use of oAB alone is comparable and not inferior to the combination of preoperative MBP plus oAB [34,35].

In 2007, Contant et al. proposed, through a multicenter randomized trial, that the use of MBP before elective CRC surgery does not significantly reduce postoperative complications, thereby suggesting its safe abandonment [36]. Subsequent randomized trials and meta-analyses reinforced and elaborated on this suggestion [37,38,39]. Leenen et al., Rollins et al. and Lewis et al. provided no significant evidence supporting the efficacy of MBP in reducing postoperative complications for elective CRC surgery. However, they acknowledge the need for further investigation, particularly for patients undergoing rectal surgeries below the peritoneal reflection and minimally invasive procedures and those with restored bowel continuity [40,41,42]. Recent research has shifted its focus to the preoperative use of antibiotics, either orally or IV, without the inclusion of MBP. Multiple studies indicate that the incidence of postoperative complications, such as ALs and SSIs, remains unchanged when using preoperative antibiotic bowel preparation, regardless of whether it is administered with or without MBP. These findings prompt a reassessment of the standard preoperative regimen and suggest that preoperative oAB alone should be considered as the new standard of care [43,44,45,46,47].

The present study has certain limitations, such as the per-protocol analysis, which elevates the potential for method-related bias. In addition, its sample size of 216 patients is adequate, but the post hoc power analysis (52%) revealed that a larger sample would result in more confident conclusions. However, due to COVID-19 restrictions, the sample size could not be further increased. On the other hand, our randomized trial presents important advantages. Its double-blind design diminishes the risk of observer-related bias, while the block randomization method eradicates confounding factors. Another notable strength of the present study lies in the trial’s prospective character, complemented by the evident homogeneity of the collected data. Finally, this is the first randomized study in the literature that investigates the use of preoperative oral administration of rifaximin in patients undergoing elective surgery due to colorectal cancer, although retrospective studies investigating the use of preoperative rifaximin administration have been performed [48]. Rifaximin is a costly and widely available antibiotic agent with low toxicity, whose clinical significance could lead to an upgrade of healthcare level even in developing countries.

Our finding of decreased SSIs after the preoperative administration of MBP plus oAB supports the implementation of this approach as a standard protocol in elective CRC surgeries. Surgeons and health care providers could incorporate this evidence-based practice into their preoperative procedures to reduce the risk of subsequent SSI. This may involve updating clinical protocols to reflect the proven efficacy of this intervention. Furthermore, there is considerable ground to cover in future research. Firstly, the investigation of the optimal preoperative oAB regimen must be investigated [49]. Our study used metronidazole in conjunction with rifaximin for the first time in the literature, the latter being known for its positive modulation of gut microbiota [50]. In addition, there is a need to explore the duration and route of administration for preoperative AB. Current literature debates on the potential superiority of a combined oral and parenteral approach compared with the parenteral route alone [51,52,53]. A personalized strategy employing risk stratification would prove beneficial. This approach could involve the tailoring of preoperative oAB in conjunction with MBP based on patient-specific factors, including comorbidities, immune status, microbiome characteristics and laparoscopic versus open CRC surgery [54,55].

## 5. Conclusions

Complications after elective surgery due to colorectal cancer burden health systems due to prolonged hospitalization and increased healthcare costs. On the other hand, they affect the oncological outcomes of patients, as they lead to delays in receiving adjuvant treatment and the deterioration of general conditions. A debate concerning the optimal preoperative bowel preparation regimen for elective CRC surgeries is currently underway. Extensive research is being conducted to investigate the impact of MBP, antibiotics or a combination of both on the incidence of postoperative complications. Preoperative oral administration of rifaximin and metronidazole in combination with mechanical bowel preparation seems to have a significant effect in reducing postoperative SSI rates. However, it seems that it has no impact on anastomotic leak, overall complication readmission rates and length of stay. Under these circumstances, preoperative oral antibiotic administration in combination with MBP should be implemented in clinical protocols. Furthermore, the most effective regimens and the way of their administration will be indicated by future research.

## Figures and Tables

**Table 1:** Patient characteristics and postoperative outcomes.

Patient Characteristics	
Age	70 ± 11 years
Gender	
Male	118 (58%)
Female	85 (42%)
Tumor location	
Ascending colon	36 (18%)
Transverse colon	46 (22%)
Descending colon	25 (12%)
Sigmoid colon	56 (27%)
Rectum	42 (21%)
Recurrence	24 (12%)
Hypertension	100 (49%)
Diabetes	42 (21%)
Heart arrhythmia	15 (7%)
Ischemic heart disease	25 (12%)
Renal dysfunction	3 (2%)
Chronic obstructive pulmonary disease	17 (8%)
Other	32 (16%)
Past surgery	103 (50%)
Postoperative outcomes	
Surgical site infection	24 (12%)
Anastomotic leakage	11 (5%)
Overall complications	46 (22%)
Readmission	15 (7%)
Length of stay (>7 days)	155 (76%)

**Table 2:** Association of preoperative oral antibiotic administration with postoperative outcomes.

	Group		*p*-Value
	Intervention	Control	
Surgical Site Infection	7 (7%)	17 (16%)	0.049
Anastomotic Leak	4 (4%)	7 (7%)	0.447
Overall complications	17 (18%)	29 (28%)	0.095
Readmission	5 (5%)	10 (10%)	0.237
Length of stay (>7 days)	75 (77%)	80 (76%)	0.955

## Data Availability

The data that support the findings of this study are available from the corresponding author upon reasonable request.
